# INTRODUCTION: SUSTAINING THE LIFE OF THE POLIS

**DOI:** 10.1017/S0001972013000430

**Published:** 2013-11

**Authors:** P. Wenzel Geissler, Ann H. Kelly, John Manton, Ruth J. Prince, Noémi Tousignant

## Abstract

How are publics of protection and care defined in African cities today? The effects of globalization and neo-liberal policies on urban space are well documented. From London to São Paulo, denationalization, privatization, offshoring and cuts in state expenditure are creating enclaves and exclusions, resulting in fragmented, stratified social geographies (see Caldeira 2000; Ong 2006; Harvey 2006; Murray 2011). ‘Networked archipelagoes’, islands connected by transnational circulations of capital, displace other spatial relations and imaginaries. Spaces of encompassment, especially, such as ‘the nation’ or simply ‘society’ as defined by inclusion within a whole, lose practical value and intellectual purchase as referents of citizenship (Gupta and Ferguson 2002; Ferguson 2005). In African cities, where humanitarian, experimental or market logics dominate the distribution of sanitation and healthcare, this fragmentation is particularly stark (see, for example, Redfield 2006, 2012; Fassin 2007; Bredeloup *et al*. 2008; Nguyen 2012). Privilege and crisis interrupt older contiguities, delineating spaces and times of exception. The ‘public’ of health is defined by survival or consumption, obscuring the human as bearer of civic rights and responsibilities, as inhabitants of ‘objective’ material worlds ‘common to all of us’ (Arendt 1958: 52). Is it possible, under these conditions, to enact and imagine public health as a project of citizens, animated in civic space?

How are publics of protection and care defined in African cities today? The effects of globalization and neo-liberal policies on urban space are well documented. From London to São Paulo, denationalization, privatization, offshoring and cuts in state expenditure are creating enclaves and exclusions, resulting in fragmented, stratified social geographies (see Caldeira 2000; Ong 2006; Harvey 2006; Murray 2011). ‘Networked archipelagoes’, islands connected by transnational circulations of capital, displace other spatial relations and imaginaries. Spaces of encompassment, especially, such as ‘the nation’ or simply ‘society’ as defined by inclusion within a whole, lose practical value and intellectual purchase as referents of citizenship (Gupta and Ferguson 2002; Ferguson 2005). In African cities, where humanitarian, experimental or market logics dominate the distribution of sanitation and healthcare, this fragmentation is particularly stark (see, for example, Redfield 2006, 2012; Fassin 2007; Bredeloup *et al*. 2008; Nguyen 2012). Privilege and crisis interrupt older contiguities, delineating spaces and times of exception. The ‘public’ of health is defined by survival or consumption, obscuring the human as bearer of civic rights and responsibilities, as inhabitants of ‘objective’ material worlds ‘common to all of us’ (Arendt 1958: 52). Is it possible, under these conditions, to enact and imagine public health as a project of citizens, animated in civic space?

## PUBLIC HEALTH, AT STREET LEVEL

The essays below bring into view workers at the lower levels of urban medical research, sanitation, healthcare and public health; a group of African citizens who, historically, have had a well-defined set of rights and responsibilities for their civic space. Moving across the city to collect rubbish, data, samples and patient follow-up forms, they engage directly with the urban population or segments of it. We followed this movement: its fluidity and abruptness, its locations and delineations, its crossings and stoppages, the materials it touches upon, and the boundaries it traces and transgresses. Guided by professional tasks and personal aspirations, interrupted by obstacles and gaps – in employment, infrastructure or supplies – they deploy their skills and labour to serve and thereby recognize a public of fellow citizens.

We call them ‘street-level health workers’, invoking Michael Lipsky's (1980) ‘street-level bureaucrats’; those who inhabit and manipulate the interface between official structures and citizens' lives, between the planners' offices and the public spaces of governance. But can they still be called bureaucrats? As volunteers, thesis students, uniformed cadets, community participants or employees on short-term contracts, they mediate between institutions and citizens, but not in a smooth and sustained way over the course of their work lives. Their positions in NGOs, research partnerships, municipal politics and public health regulation are uncertain, usually temporary and often informal. They no longer embody the figure of the street-level worker as a routine labourer maintaining the city as part of a stable career. This older figure, at least potentially, could acquire expertise, a sense of vocation and of cumulative transformative action on health, over the course of her/his life, linking the material *urbs* to the civic *polis*. But what happens to these ties – between individual and collective, daily repetition and better future, *urbs* and *polis* – when the duration, institutional attachments and material conditions of street-level work are eroded or destabilized?

Rapid population growth and the privatization of ‘public’ services, including water provision, sanitation and healthcare, are major challenges to urban public health in Africa. Secure jobs and especially public employment are increasingly rare for public health workers in Ibadan, Dakar and Kisumu. They live in and care for expanding cities dominated by informal economies and planning, positioning themselves as observers but also inhabitants of unregulated spaces: from slums and unplanned urban developments to illicit drug markets. Yet recent changes in the healthcare landscape have also afforded new opportunities that are particularly marked in, if not unique to, each of these cities. Kisumu's growth in the past fifteen years is clearly associated with transnational investments in HIV research and care. This ‘HIV economy’ generates clinical trials and NGO activity, but also new spaces and relations of housing and leisure, prevention and care, affecting definitions of ‘community’ and ‘employment’ and, more generally, modes of living and advancing in the city. Two essays in this collection describe the promises and uncertainties associated with health work in this economy. In Dakar, health and sanitation are materialized in the urban landscape as hundreds of private clinics and pharmacies, ambulance services, doctors' and dentists' offices, health insurance companies and garbage trucks bearing company names and logos. Along with visible growth in urban middle-class housing and consumption, this thriving healthcare market redraws lines of inclusion and exclusion in the city, generating new subjectivities of health work. The interpenetration of municipal enterprise and market activity in sanitation in Ibadan reflects patterns of spatial exclusion and inequalities in exposure to environmental risk, but also a long history of elite political struggles for the city, as slogans are projected and erased on t-shirts, Hilux trucks and garbage disposal vehicles. The tangibility of political and paramilitary interference is made manifest at monthly sanitary checkpoints, policing movement around the city as sanitary policies are at once performed and traduced.

The essays below describe how public health workers engage with similar challenges and differentiated opportunities resulting from the disinvestment of the state in systems of healthcare and sanitation – from privatization and NGO-ization, to transnational research and forms of political performance. But we are also interested in how these cities, despite differences in their histories, are shaped by broadly similar patterns associated with colonialism and other forms of integration in the global economy, including segregation and development, decline and differentiation, and urban–rural migration. The city has long been seen as a laboratory of civic subjectivity. It has become a space for making claims, taking responsibility and imagining communities that are both smaller and larger than the nation (Holston and Appadurai 1996; Simone 2001; Gupta and Ferguson 2002; Massey 2004; Harvey 2006; Sassen 2006). Yet the city not only escapes and supersedes but also retains vestiges of the ‘the national as container of social process and power’ (Sassen 2003: 57). The African city is a rapidly changing entity, but also an archive of colonial and national projects of inclusion and exclusion, retaining marks of their spatiality (of segregation and radiation) as well as their temporalities (of routines and progress) (Winters 1982; Slaughter 2004; Mbembe and Nuttall 2008).

Even in workers' momentary engagement with public, civic and urban health as we analyse it here (the research theses of pharmacy students, ‘Sanitation Day’, the volunteer-provided HIV/AIDS education), and within its limited reach (the checkpoint, the trial community, the ‘slum’), we encounter aspirations to and imaginations of progress. Workers move across the city within their workdays but also, as they pressed us to notice, over the larger timeframes of the city's histories and of their own work lives. Their movements thus extend to former civic capacities and future possibilities, invoking more-than-urban (local, national, transnational) frames of action, exchange and obligation. While our informants act on a space that is increasingly folded onto itself and/or connected to distant ‘global’ spaces, they also, in different ways, challenge and refuse this partition and involution of the city by remembering and reaching towards more comprehensive collectives and solidarities. Despite the new uncertainties and opportunities they face, the workers we studied retain or refer to elements of street-level mobility, mediation and civic commitment that characterize older groups of civil servants, inviting us to study their histories and those of previous street-level workers.

## STREET-LEVEL HISTORIES

Colonial administrations relied on African intermediaries in public health and sanitation, increasing their numbers from the 1940s to serve new policies of development (see, for example, Packard 1997; Iliffe 1998; Lawrance *et al*. 2006). During the post-independence decades (1960s–1970s), in the context of health sector expansion and primary healthcare policies, nationalist governments often expanded these services and pursued the ‘Africanization’ of all levels of public health workers (see, for example, Hunt 1999). Cities and public health figured centrally in the ambitious but incremental projects of new nation states for societal transformation and citizenship formation (see Ferguson 1999; Slaughter 2004).

Post-colonial governments and their street-level health workers inherited and acted upon contradictory legacies of urbanization, modernization and public health. These included amelioration and development, with cities animated by visions of scientific progress and modern order, written in the layouts of streets and neighbourhoods, and taking shape in the design and location of hospitals, laboratories and medical universities (see, for example, Curtin 1985). Concentrated in cities, these institutions not only served the needs of an expatriate population, but also showcased the capacity and long-sightedness of colonial governments. Yet African cities, before and after independence, were created as healthy and modern through violent means (Collignon 1984; Echenberg 2002). The status of being urban (and thus modern) was denied to most Africans, whether residing in cities or not (see, for example, Packard 1989; Mamdani 1996; Georg 2006).

At least during their first decades, new nation states were able to sustain urban health work as a stable job. This opened the possibility of tying the mundane routines of street-level work across urban space (and over days and lives) to expansive civic imaginaries – reaching out towards national spaces and futures – that are remarkably similar across radically different political ideologies and aspirations. If precarious employment challenges such ways of marking and making the city, today's street-level workers encounter the traces of these more continuous and certain trajectories. Despite the ambiguities of post-independence public health, and perhaps because it was so ephemeral, it remains today in yearning for a more robust public health, and many Africans look back on this period with nostalgia for the visions of an expansive and inclusive progress it endorsed (Prince and Marsland 2013).

From the mid-1970s, such trajectories were disrupted by the combined effects of economic crisis, austerity measures and the dwindling legitimacy of most African states, precipitating a period, lasting into the 1990s, of government retrenchment and decline (see, for example, Olowu 2001). The incapacity of states to propel economic development made the political project of sovereignty increasingly untenable, reducing the scope of political visions. Budget cuts affected the functioning of government institutions: hiring slowed or stopped, staff and supplies were short, infrastructures decayed and, in some cases, salaries were reduced, delayed or unpaid. The ‘emptying out’ of many public facilities and services (for example, in clinics that lacked medicines or doctors) diminished populations' as well as civil servants' expectations of the potential of state action for social transformation, as well as for social control (Masquelier 2001). Government agents such as street-level health workers lacked the means – vehicles, lab reagents, syringes – to move across and act on urban space (see, for example, Geissler 2011), affecting the reach, and thus the expectations and aspirations, associated with public health and other civic projects. Many moved into or expanded their private sector activities. The materiality and epidemiology of cities were also transformed by lack of public regulation and maintenance; the growth of the informal sector and the poor condition of roads and sewers eroded a sense of public responsibility for order and health (see Sanders 2008).

Some of the strategies deployed to fill the gaps left by government contraction have created new openings for health work. From about the mid-1990s, and especially in the past decade, these openings have been exploited by a growing number of commercial, non-governmental and transnational organizations. These hire technical and field staff to extend their reach, but often on a short-term basis, and for demographically and epidemiologically circumscribed projects. Our collection of articles observes how street-level health workers engage with their work and cities in multiple ways, including but not limited to these emerging tasks and opportunities, but also through pasts of emergence and decline, towards projects, publics and futures beyond the horizons of current configurations of labour and health. Our analysis seizes workers' memories and encounters with vestiges of older ways of moving and staying in the city to show how public health work produces different lives and cities than it did in the past. But we also show how workers' ‘future orientations’ (Guyer 2007) evoke other possible material forms, social textures and spatial extensions their work might take. They comment on losing but also gaining futures reached by forward-moving, rather than regressive or stagnant pathways, imagining multiple territorialities, and thus communities they might act upon. They thus raise questions not only about who were, are and should be the publics of public health (Kelly and MacGregor, in press; Prince and Marsland 2013), but also about how specific forms of labour, expertise and vocation might create and affiliate such publics. Thus our attention to the changing spatial and temporal forms of street-level health work leads us from the city to the polis, turning ethnographies of urban public health into ethnographies that explore the ambiguous arena of civic commitment.

## ASPIRING BEYOND RUPTURE

In Wenzel Geissler's article on two generations of Kenyan scientific fieldworkers, the author's mapping of research institutions, changing urban textures, fieldwork circulations and health workers' residential patterns reveals a progressive fragmentation of the cityscape. Earlier centre–periphery patterns, and attendant processes of extension, exploration and colonization of urban territories, are replaced with intersecting archipelagoes. Urban public health extension work that used to radiate across all the city's sectors is replaced with fragmented non-governmental interventions focusing on high-density, poor areas. Yet, in spite of the seemingly radically different geography of this public health archipelago, Geissler also traces geographical persistence, lasting memories of older urban health, and affective continuities in the health workers' lives, which anchor the present in the past while ultimately underscoring an overall sense of rupture.

The young pharmacists studied by Noémi Tousignant and the NGO volunteers studied by Ruth Prince locate street-level health work within the course of their lives. They reach for identities and forms of labour that evoke professional careers, social mobility and figures of success. By collecting data for their pharmacy theses, or doing HIV outreach work, they also enact a ‘population’ or ‘community’ that makes up the city as an inclusive space of public health action. Yet this enactment is ephemeral; without continuous repetition over the long term, it does not lead to durable transformations of the city as a civic space or to stable employment for themselves as civic actors. Instead, pharmacy students expect to work in the private sector, while volunteers might end up ‘tarmacking’ (looking for work) or stringing together short-term attachments to health NGOs. Their trajectories may thus seem flat and futureless. Yet both Tousignant and Prince note how these individual trajectories acquire a sense of forward projection as students and volunteers juxtapose past, present and potential ways of moving around the city.

Finally, John Manton asks how the event of Environmental Sanitation Day relates to the broader movements, spatiality, histories and temporalities of urban sanitation in Ibadan. As a public spectacle, this repeated half-day occurrence is concentrated in the space of the checkpoint. Although static and short, the checkpoint is a site of both performance and resistance to the projection of urban sanitation across the topographical and (imperfectly) class-stratified zones of the contemporary Nigerian city. Its staff, comprising state-employed environmental health officers (EHOs) and uniformed voluntary marshals and cadets, struggle to extend practices of sanitation across urban space. Yet the described movement of gleaming trucks bearing electoral slogans reveals the fragmentation and politicization of rubbish removal that operates largely in wealthier parts of the city. Animated by their own sense of civic responsibility and the goal of urging urban residents to take responsibility for their streetscape, EHOs and paramilitary volunteers instead become performers of ‘the simulacrum of responsible governance’.

All four articles relate current temporal and spatial configurations of street-level health work to broader transformations of urban life and public health. They attempt to grasp both the past-in-the-present and future orientations. Tousignant and Geissler invoke this through explicit generational contrast: young health workers attached to transnational research in Kenya and pharmacy students in Senegal face different configurations of work compared to the post-independence generation of public health workers, yet their everyday labour, movements and dreams retain traces of these pasts. Prince's volunteers do not so much yearn for the past as turn their back on it as they embrace what they perceive to be new opportunities and possibilities that arrive with ‘global health’. Manton depicts an uneasy retreat from historical and ideal visions of the healthy post-colonial city and the public good, as the sanitary functions of environmental health workers are displaced amid urban politics and the elaboration of a coercive rent-seeking apparatus of ‘inspection’.

All four articles also invite us to think through how personal aspirations have become connected to, or disconnected from, imaginations and enactments of the public good. The experience of living and working in the city can engender a sense of responsibility towards a collective and aspirations concerning progress, while simultaneously encouraging disengagement and cynicism.
